# Correlation of cellular traction forces and dissociation kinetics of adhesive protein zyxin revealed by multi-parametric live cell microscopy

**DOI:** 10.1371/journal.pone.0251411

**Published:** 2021-05-11

**Authors:** Lorena Sigaut, Micaela Bianchi, Catalina von Bilderling, Lía Isabel Pietrasanta

**Affiliations:** 1 Departamento de Física and Instituto de Física de Buenos Aires (IFIBA), CONICET-UBA, Facultad de Ciencias Exactas y Naturales, Universidad de Buenos Aires, Buenos Aires, Argentina; 2 Consejo Nacional de Investigaciones Científicas y Técnicas (CONICET), Buenos Aires, Argentina; 3 Centro de Microscopías Avanzadas, Facultad de Ciencias Exactas y Naturales, Universidad de Buenos Aires, Buenos Aires, Argentina; 4 Instituto de Investigaciones Fisicoquímicas Teóricas y Aplicadas (INIFTA), UNLP-CONICET, La Plata, Buenos Aires, Argentina; 5 Departamento de Física, Facultad de Ciencias Exactas y Naturales, Universidad de Buenos Aires, Buenos Aires, Argentina; Oregon State University, UNITED STATES

## Abstract

Cells exert traction forces on the extracellular matrix to which they are adhered through the formation of focal adhesions. Spatial-temporal regulation of traction forces is crucial in cell adhesion, migration, cellular division, and remodeling of the extracellular matrix. By cultivating cells on polyacrylamide hydrogels of different stiffness we were able to investigate the effects of substrate stiffness on the generation of cellular traction forces by Traction Force Microscopy (TFM), and characterize the molecular dynamics of the focal adhesion protein zyxin by Fluorescence Correlation Spectroscopy (FCS) and Fluorescence Recovery After Photobleaching (FRAP). As the rigidity of the substrate increases, we observed an increment of both, cellular traction generation and zyxin residence time at the focal adhesions, while its diffusion would not be altered. Moreover, we found a positive correlation between the traction forces exerted by cells and the residence time of zyxin at the substrate elasticities studied. We found that this correlation persists at the subcellular level, even if there is no variation in substrate stiffness, revealing that focal adhesions that exert greater traction present longer residence time for zyxin, i.e., zyxin protein has less probability to dissociate from the focal adhesion.

## Introduction

Cells detect, process and translate the mechanical information that is provided by the extracellular environment to make decisions about growth, motility and differentiation [1]. In particular, the stiffness of the microenvironment impacts in cell spreading [2], cell morphology, cytoskeletal organization and contractility, and stem cell differentiation [3]. For instance, cells can respond to their microenvironment by changing mechanical properties, such as cell elasticity modulus, which is altered in its transformation from normal to malignant within the tumor’s microenvironment in cancer progression [4]. Cells actively sense the rigidity of their surroundings, exerting traction forces through the focal adhesions, large multiprotein structures that mechanically connect cytoskeleton components with the extracellular matrix, through the integrin membrane receptors. Focal adhesions are highly dynamic structures that exhibit mechanosensitive properties acting, not only as traction points, but also as signaling organelles in the cell mechanotransduction process [5]. Many of the molecules that compose them are proposed to interact with one another to form an integrated mechanical and biochemical network that regulates the processes involved in cell adhesion and substrate sensing. In particular, we are interested in one of the key focal adhesion proteins, zyxin, postulated as a mechanosensor protein. By interacting with a great variety of cellular proteins, it is known to contribute to fundamental cellular functions such as cell migration and adhesion, having a leading role in cellular response to mechanical cues. Zyxin protein is recruited at the final stages of the focal adhesion assembly [6], and its binding kinetics is known to be influenced by mechanical forces. Previous findings have shown that zyxin unbinding kinetics is altered by substrate stiffness [7], by mechanical disruption or pharmacological inhibition of cytoskeletal tension [7–9] and by increasing cellular tension by mechanically stretching cells [9].

To study cellular responses to substrate stiffness, polyacrylamide hydrogels (PAA) are one of the most commonly used substrate materials, due to their mechanical tunability, optical translucency, and elastic material behavior. By controlling the relative fraction of their constituents, polyacrylamide hydrogel of varying Young’s modulus can be obtained [10]. Moreover, fiducial markers can be embedded into these hydrogels to be used as substrate for quantitative measurements of cellular traction forces, in traction force microscopy (TFM). TFM is a powerful and widely used tool that provides spatially-resolved measurements of the direction and magnitude of cellular traction forces on an elastic substrate through the quantification of its deformation [11]. It has been seen in previous studies using deformable gels [12] or micropost arrays [13, 14], that substrate stiffness can strongly influence the generation of cellular traction forces, indicating that cellular traction forces increase in cells cultivated at increasing substrate rigidity. The stiffness of the microenvironment also affects the magnitude of forces generated by metastatic and non-metastatic cells [15], suggesting that metastatic cells are able to exert greater net traction forces than non-metastatic cells. It was also shown that substrate rigidity increases cellular traction forces and enhances epithelial ovarian cancer cell migration [16].

Focal adhesion dynamics and cellular traction forces are closely related and are both involved in substrate rigidity sensing. Therefore, the ability to measure cellular traction forces together with quantifying focal adhesion molecular dynamics is essential to better understand the molecular mechanisms behind substrate mechanosensing. In this work, we present an approach based on a combination of several microscopies and quantitative data analysis that allowed us to explore the correlation between the generation of traction forces and zyxin dynamics at focal adhesions, by combining live cell imaging, traction force microscopy, advanced fluorescence techniques such as fluorescence recovery after photobleaching (FRAP) and fluorescence correlation spectroscopy (FCS), in addition to the fabrication of adjustable stiffness polyacrylamide hydrogels and the characterization of their elasticity by force spectroscopy using an atomic force microscope. By cultivating cells on polyacrylamide hydrogels of different stiffness we were able to investigate the effects of substrate stiffness on the generation of cellular traction forces by TFM and characterized the molecular dynamics of the focal adhesion protein zyxin, by FCS and FRAP. As the rigidity of the substrate increases, we observed an increment of both, traction force generation and zyxin residence time at the focal adhesion, while there was no appreciable change in its diffusion. Moreover, at the studied substrate elasticities we found a positive correlation between the traction forces exerted by cells and the residence time of zyxin at focal adhesion. To further explore this correlation, we performed combined TFM and FRAP experiments. Even though there were no variations in substrate stiffness (combined experiments are performed in the same cell), we observed a linear correlation–at the subcellular level- between the zyxin residence time and the magnitude of the traction forces exerted by focal adhesions revealing that regions of focal adhesions that exert greater traction forces, present smaller dissociation rate constants for zyxin. Previous works have suggested a relation between mechanical stimulus and zyxin dynamics [7, 8]. However, no experimental quantitative evidence of the direct relation between measured cellular traction forces and zyxin dynamics at focal adhesions was reported. A key advantage in the approach presented here lies in the possibility of an integral and multiparametric single cell analysis. This approach allows us to establish a direct correlation between cellular traction force and zyxin molecular dynamics which, to our knowledge, was not established before. These results provide further evidence reinforcing the mechanosensitive properties of zyxin, pointing it out as a key protein for cellular traction forces.

## Results

### Polyacrylamide hydrogels exhibit reproducible and adjustable stiffness values

In order to study the effects of the elasticity of the substrate in the generation of forces and in the dynamics of the focal adhesion protein zyxin in mouse mammary epithelial living cells, we need a reproducible and reliable way to obtain substrates with different mechanical properties. To this end, we use PAA hydrogels due to their easily controlled mechanical properties, elastic material behavior and optical transparency. Its main components are acrylamide and bis-acrylamide, which are catalyzed by ammonium persulfate and N,N,N’,N’-tetramethylethylenediamine (TEMED) to form the hydrogel. Depending on the mole fraction of acrylamide and bis-acrylamide, substrates with different elasticity can be obtained. According to the cell line studied, there is a range of elasticities for which TFM can be applied; the substrate must be soft enough for the cell to deform it and sufficiently stiff so the cells can adhere and form focal adhesions. We tested different concentrations of acrylamide and bis-acrylamide based on the results published by Aratyn-Schaus *et al*., 2010 [10], and found that ranges of 0.05% to 0.28% bis-acrylamide and 7.5% to 12% acrylamide (Young’s modulus between 3.4 kPa and 37 kPa) were the most suitable for mouse mammary epithelial HC11 cells (see Table 1). In this range of elasticity, cells are well adhered to the substrate and are able to form focal adhesions. We performed TFM experiments in a more rigid substrate (Young’s modulus of 46 kPa) but no appreciable substrate deformations were detected, so no estimation of the traction force was possible. This would be an upper limit of substrate rigidity for TFM experiments in mouse mammary epithelial HC11 cells.

**Table 1:** Composition of acrylamide/bis-acrylamide of fabricated polyacrylamide substrates.

PAA hydrogel	Acrylamide (%)	Bis-Acrylamide (%)	N_exp_	*E* (kPa)
A	7.5	0.05	-	3.4 *
B	7.5	0.3	6	13 ± 1
C	12	0.14	7	18 ± 1
D	12	0.28	4	37 ± 7
E	12	0.6	6	46 ± 4

Young’s modulus, *E*, measured averaging the results of force-distance curves obtained in N_exp_ experiments. More than 1000 curves were analyzed for each PAA composition. Errors represent the standard error (SE).

* estimated by relative concentrations, using as a reference values obtained in Aratyn-Schaus *et al*., 2010 [10] and the measured *E* of hydrogel B.

Having a reliable value of the Young’s modulus is necessary to reconstruct the traction forces exerted by the cell on the substrate. The PAA hydrogel Young’s modulus can be estimated from the concentrations of the components used in its fabrication, but to have a more accurate value it is preferable to measure it. To this end, we characterized mechanical properties of the PAA substrates by force spectroscopy using an atomic force microscope. The substrate Young’s modulus was determined from the analysis of the approach force-distance curves, employing the Sneddon model (see the Materials and methods section for further details). Independent force spectroscopy experiments yielded consistent values of PAA hydrogel Young’s modulus. The obtained values are shown in Table 1, and representative force curves are presented in S1 Fig. The softest studied PAA hydrogel (hydrogel A) was too soft to acquire reliable force-distance curves, so the Young’s modulus was estimated by construction, using as a reference the measured Young’s modulus value of hydrogel B.

As expected, we observed that the measured PAA hydrogels Young’s modulus scales with the amount of the crosslinker, bis-acrylamide, according to data published in the literature [10, 17]. This linear behavior was previously found in hydrogels fabricated with a wide range of concentrations of bis-acrylamide (0.03% to 0.3% w/v) and different percentages of acrylamide (3% to 10%), by using force spectroscopy and employing force sensors with spherical or pyramidal geometries [17].

### Cellular traction generation is modulated by substrate elasticity

To analyze the effect of the substrate elasticity on the generation of cellular traction forces, we performed TFM experiments in HC11 cells cultivated on fibronectin-coated polyacrylamide hydrogels of different Young’s modulus (3.4 kPa, 13 kPa, 37 kPa and 46 kPa) containing 40 nm fluorescent nanospheres as reference markers. To quantify the elastic deformation of the substrate and cellular tractions induced by a single cell, it is necessary to find an isolated cell, that is, cells must be sufficiently separated so that the deformations generated by one cell do not interfere with those of another. Once a cell of interest is found, the distribution of the fluorescent markers is recorded to show the derformed substrate. Then cells are detached from the substrate by trypsinization and a second image is acquired to serve as a reference of the undeformed substrate. From these images, the positions of the markers were tracked using the particle image velocimetry (PIV) algorithm employing the open-source Matlab code MatPIV V1.6.1 [18]. This algorithm is based on cross-correlating image sub-regions between sequential pairs of images. The deformation map shows in each window the vector of the deformation obtained by locating the peaks of the cross correlation between the PRE and POST images of each interrogation window. The color code represents the module of the intensity of the deformation.

Once the deformation field is obtained, it is necessary to know some elastic parameters of the substrate for the traction force reconstruction, such as its Young’s modulus and the value of the Poisson’s ratio, as we assume PAA hydrogels are an elastic, isotropic and semi-infinite material. The hydrogel is considered an incompressible material, its Poisson’s ratio value being equal to 0.5, and in order to have a reliable value, Young’s modulus was determined experimentally by force spectroscopy (Table 1). The traction field was obtained by applying Fourier transform traction cytometry (FTTC) [19]. Since being a poorly conditioned inverse problem, highly sensitive to deformation errors, we employed the Tikhonov regularization method [20], incorporating a regularization parameter. To select the optimal value of the regularization parameter we use the L-Curve criterion [21] (see Materials and methods).

**Fig 1:**
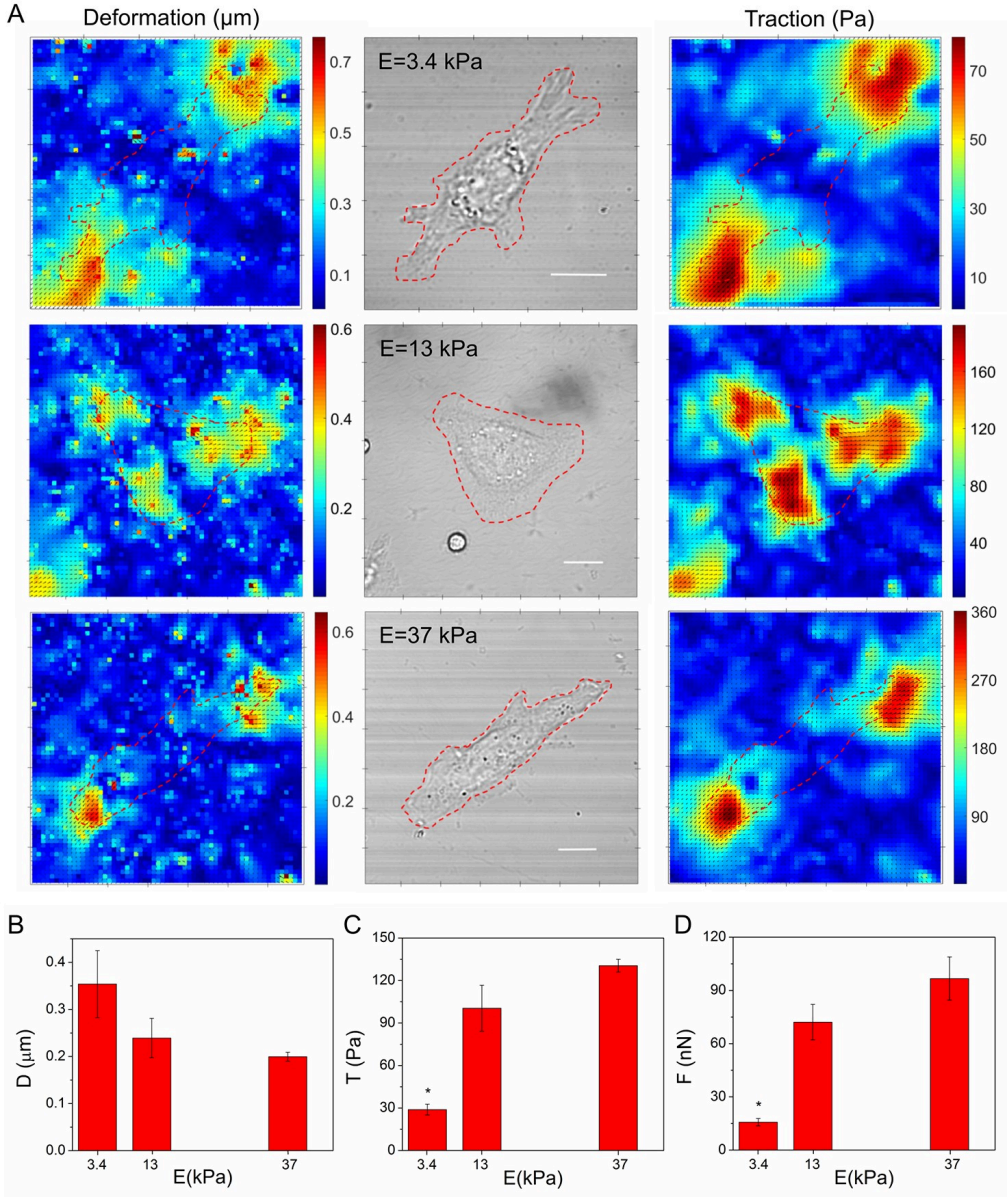
Cellular traction forces of cells cultivated on substrates of different stiffness. **(A)** Representative deformation (left column) and traction maps (right column) for HC11 cells grown on PAA substrates of different Young’s modulus (3.4 kPa, 13 kPa and 37 kPa). The border of each cell is delimited by red dashed lines in each image. Transmission images (center column), scale bars 10 μm. The average deformation *D* **(B)**, traction *T* **(C),** and force *F* **(D)** magnitudes generated by HC11 cells grown on PAA substrates of different elasticity (N = 4, N = 8, N = 2 cells for hydrogels of 3.4 kPa, 13 kPa and 37 kPa, respectively; bars are the SE error). The values of deformation, traction and force were analyzed by the MannWhitney test, the populations that are significantly different from each other (P<0.05) are indicated with *.

For the elasticities studied we present in Fig 1A representative deformation and traction maps together with the cell transmission image. As it can be seen in the transmission images presented in Fig 1A, cells are completely extended and well adhered, so substrate deformations are mainly contained in the plane and normal forces to the substrate surface are minimized. Also, TFM experiments were performed in isolated cells, so there are no deformations due to surrounding neighbours cells and only the deformation due to the studied cell is quantified. It can be appreciated in the deformation maps presented in Fig 1A that, no matter the elasticity of the substrate, deformations are located preferencially at the periphery of the cell where focal adhesions are more frequent. It can also be distinguished in Fig 1A that the generated cellular tractions increase as the substrate becomes more rigid. As mentioned, to estimate cellular tractions, the substrate is considered as an elastic, isotropic and semi-infinite material. The PAA hydrogels can be considered elastic for small deformations with respect to the spatial scale of deformation variation, which in this case is determined by the characteristic size of the focal adhesions. Since the typical size of focal adhesion is about 1 μm, and for all the elasticities studied, the maximum deformations are smaller than 1 μm, this hypothesis is satisfied. Moreover, as the hydrogel thickness (~50 μm) is much greater than the deformations, the hydrogel can be considered as a semi-infinite space.

It can be distinguished in the reconstructed traction maps presented in Fig 1A, that traction forces follow the deformation and are located preferentially at the periphery of the cell. Considering the traction stress color map indicated on each map, it can be seen that the generated cellular tractions increase as the substrate becomes more rigid. To have an overall view, for each TFM experiment, the total amount of deformation and traction exerted by a single cell were characterized. To this end, cellular area were delimited in the transmission images and by applying the cell mask to the deformation or traction maps, the average values of the deformation and traction exerted by the cell on each hydrogel were calculated. Then the results of the experiments performed on each substrate condition were averaged. The average values of the deformations decrease according to the increase of the Young modulus of the substrate, although no significant differences were seen, while the average cellular traction and cellular forces increase by increasing the stiffness of the substrate, and is significantly different for 3.4 kPa substrates with respect to 13 kPa and 37 kPa (Fig 1B–1D).

These results indicate that mouse mammary epithelial cells respond to increasing substrate stiffness by generating greater traction forces. These results agree with previous studies of traction force exerted by human mammary epithelial cells [15] and bovine aortic endothelial cells [12] at increasing substrate stiffness (1–10) kPa, and are in the same direction as published results employing microposts deflection to measure cell traction exerted by human pulmonary artery endothelial cells [13] and human embryonic fibroblasts [14].

### Substrate elasticity alters molecular kinetics of the focal adhesion protein zyxin

Zyxin protein is recruited at the final stages of the focal adhesion assembly [6], and may interact with a great variety of cellular proteins, having a leading role in cellular response to mechanical cues. To get a deeper insight into the effect of substrate rigidity at a focal adhesion level, we will explore the relation between the dynamics and binding kinetics of zyxin protein and the substrate elasticity.

To investigate the effects of substrate elasticity on the dynamics of zyxin, we characterized the molecular diffusion of zyxin protein in living cells exploiting the temporal scales of single-point fluorescence correlation spectroscopy (pointFCS). PointFCS experiments were carried out in HC11 cells expressing zyxin-EGFP, cultivated on 13 kPa PAA substrates and on glass coverslips as a rigid substrate (elasticity ~ 70 GPa). Temporal fluorescence fluctuations in a small volume generated by a focused laser beam within a focal adhesion were recorded and the auto-correlation function was calculated. For each substrate condition, the average auto-correlation curves were fitted by 1- or 2- component model (Eq 3), and one or two effective diffusion coefficients were estimated. Since zyxin molecules may, not only diffuse, but also interact with other proteins and bind to the focal adhesion complex, an effective diffusion coefficient is obtained from FCS analysis. In the 2- component model, the fast-diffusing component can be associated to diffusion in cytosolic and the slower component is associated to transient binding to immobile structures such as adhesions [22]. Correlation analysis was performed using a custom-made routine written in Matlab platform.

**Fig 2:**
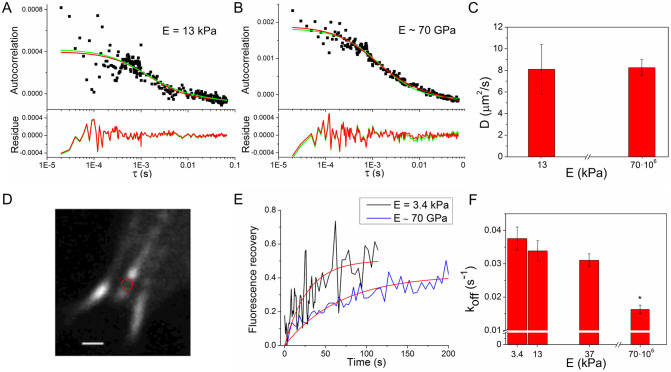
Effect of substrate stiffness on focal adhesion protein zyxin kinetics and mobility. **(A-C) Effective diffusion coefficient for zyxin evaluated by FCS**. Representative auto-correlation curves for zyxin at focal adhesions of HC11 cells grown on PAA substrate of 13 kPa **(A)** and on coverslips **(B).** In red and green lines, fitting of diffusion model of 1- or 2- components, respectively (Eq 3). Corresponding residues are shown on bottom panels. **(C)** Mean effective diffusion coefficient of zyxin in focal adhesions of cell grown on 13 kPa PAA substrate and on coverslips. Error bars represent SE. **(D-F) Zyxin unbinding kinetics in HC11 cells grown on PAA substrates evaluated by FRAP. (D)** Confocal image of a cell region with focal adhesions, scale bar 1 μm. Region selected for photobleaching within a focal adhesion is indicated with a red circle. **(E)** Representative fluorescence recovery curve for zyxin (black or blue line) and fitting (red line) by focal adhesion model Eq 7. **(F)** Mean dissociation rate constant *k*_*off*_ of zyxin as a function of substrate elasticity modulus. Last column refers to cells cultivated on glass coverslips (elasticity ~70 GPa). Error bars represent SE. Data presented in C and F, were analyzed by the MannWhitney test, the populations that are significantly different from each other (P < 0.05) are indicated with *.

Representative autocorrelation curves are presented in Fig 2A and 2B, and average effective diffusion coefficients for zyxin, together with the corresponding average amplitudes estimated for the 1- or 2- component fitting, are summarised in S1 Table. By fitting the experimental auto-correlation obtained in cells grown on the 13 kPa hydrogel using the two component model, the same effective diffusion coefficient value was obtained for both components (*D*_*1*_ *= D*_*2*_ = 11.5 μm^2^/s), and this value was the same as the one obtained from the fitting of 1-component (*D*_*1*_ = 11.5 μm^2^/s). This indicates that the correlation can be described by one representative diffusive component. In the case of cells grown on coverslips, data fitted by 1-component diffusion model, almost the same diffusion coefficient was obtained, *D* = 11.7 μm^2^/s, (Fig 2C). When fitting the data with 2-component diffusion model, there is one term corresponding to the fastest diffusion that has a similar diffusion coefficient (*D*_*1*_ = 15.8 μm^2^/s) as the one determined by 1-component fit (*D* = 11.7 μm^2^/s); and a second term with greater errors in the parameters, associated with a very slow diffusion ~30-fold slower (*D*_*2*_ = 0.34 μm^2^/s). The faster component associated with the diffusion of the protein presents a diffusion coefficient similar to the one observed for other focal adhesion proteins [22, 23], and the slower component most likely associated with binding to the focal adhesion has similar order as the one found for the protein paxillin [22].

Since the parameters estimated with the 2-component model present greater errors, and the fastest term can be associated to the 1-component model, we will consider the 1-component diffusion model to describe the correlation. Comparing the 1-component fit results for both substrates (S1 Table), there are no differences in the effective diffusion coefficients for zyxin estimated in cells grown on 13 kPa PAA hydrogels compared to cells cultivated on coverslips. Moreover, the characterization of the autocorrelation curves by one diffusive component gives diffusion coefficients for zyxin, comparable to those of other focal adhesion proteins such as vinculin, paxillin and focal adhesion kinase [23], and similar characteristic correlation time for zyxin in rat embryo fibroblast cells cultivated on glass [24].

These results would indicate that there is no appreciated effect of the elasticity of the substrate in zyxin mobility and dynamics at focal adhesion, although the signal to noise ratio could not be sufficient to appreciate differences in the effective diffusion coefficient. We will further investigate the effects of substrate on zixin molecule with another approach, to specifically address the binding kinetics, more than mobility.

Focal adhesions are highly dynamic multi-protein complexes and the dissociation rate constant, *k*_*off*_, of individual proteins from the adhesion complex is a key determinant of the turnover of focal adhesions and is proven to be sensitive to changes in applied forces [25]. In particular, molecular binding kinetics of zyxin, is known to be altered by mechanical forces. For example, manipulation of intracellular cytoskeletal structure or contractility, alters zyxin binding kinetics. It has been shown that dissipating contractile forces exerted by the actin cytoskeleton by either pharmacologically employing ROCK inhibitors or physically disrupting by laser ablation of stress fibers, resulted in an increase of the dissociation rate constant of zyxin from focal adhesions [7].

To study the effect of substrate elasticity on the molecular binding kinetics of zyxin to focal adhesions, we used an approach based on Fluorescence Recovery After Photobleaching (FRAP) [26, 27] on living HC11 cells expressing zyxin-EGFP cultivated on PAA of different elasticities. Thus, a small area within a focal adhesion was photobleached employing the confocal laser spot (Fig 2D), and the fluorescence recovery curve was monitored over time. Considering a spatially homogeneous concentration of freely diffusing molecules, temporally constant during the course of the FRAP experiment, and assuming that the diffusion of unbounded molecules is much faster than the binding kinetics, the fluorescence recovery is dominated by the dissociation of the protein from the focal adhesion [27, 28]. Under these conditions, the normalized recovery curves were fitted by Eq 7 and the unbinding constant *k*_*off*_ and mobile fraction *m* were obtained (see Materials and methods). Note that this approach provides an effective *k*_off,_ representative of all the unbinding interactions of the protein, since zyxin may interact with multiple different partners in the focal adhesion complex.

We have already corroborated in a previous work [9] the suitability of this approach to estimate zyxin *k*_*off*_ in HC11 cells cultured on coverslips, by performing FRAP experiments using the ROCK kinase inhibitor Y-27632, which dissipates cytoskeletal tension. As expected, decreasing the internal force exerted on the focal adhesions by the inhibitor treatment caused a faster fluorescent recovery of zyxin molecule into the photobleached region, i.e, caused an increase in zyxin’s *k*_*off*_.

Table 2 summarizes the results obtained with this approach, including the mobile fraction and zyxin’s residence time, corresponding to the inverse of *k*_off._ Fig 2E shows representative normalized recovery curves obtained at 3.4 kPa and coverslips substrate, which evidence a faster fluorescence recovery of zyxin in the photobleached regions for a more rigid substrate, such as glass. Fig 2F presents the average values of the unbinding constant of zyxin at the different mechanical conditions studied, showing that the dissociation constant of zyxin diminishes at increasing substrate elasticities and is significantly smaller compared to the observed in cells grown on a rigid substrate as fibronectin coated coverslips. The same tendency of decreasing *k*_*off*_ values was found in bovine adrenal capillary endothelial cells cultivated on substrates of 50 kPa compared to 300 kPa [7]. If a wider range of PAA substrate stiffness would have been studied (e.g., 3.4kPa–300kPa), the differences in zyxin kinetics could have been significant, as it was seen in Lele et al. 2006 [7]. Since HC11 cells were not found to deform substrates of 46kPa or stiffer, a physiological relevant range of PAA stiffness (3.4kPa–37kPa) was chosen, in which TFM can be performed in order to investigate the correlation between cellular traction and zyxin dynamics. The average mobile fraction, *m*, did not presented an evident tendency, having similar values for the different studied conditions except for PAA of 13 kPa which had a slightly higher value.

**Table 2:** Zyxin unbinding kinetics evaluated by FRAP in HC11 cells cultivated on substrates of different stiffness.

Substrate	N	k_off_ (1/s)	*t*_*res*_ (s)	*m*
3.4 kPa PAA	17	0.038 ± 0.003	27 ± 2	0.44 ± 0.03
13 kPa PAA	18	0.034 ± 0.003	30 ± 3	0.57 ± 0.03
37 kPa PAA	9	0.031 ± 0.002	32 ± 2	0.38 ± 0.04
Coverslips ~70 GPa*	12	0.016 ± 0.001	61 ± 5	0.41 ± 0.04

Average unbinding constant for zyxin, *k*_*off*_, and mobile fraction, *m*, determined by fitting FRAP recovery curve by Eq 7, obtained from N experiments. The characteristic time, *t*_*res*_, was obtained from *k*_*off*_ (Eq 8). Errors are SE.

* Data reported in a previous work, Sigaut *et al*., 2018 [9].

Recently, in Sigaut *et al*., 2018 [9] we have reported, employing a mechanical stretching device, that in living HC11 cells the zyxin dissociation from the focal adhesions is significantly faster for cells under low normal strain compared to cells under higher strain or grown on coverslips. We have also found that increasing the normal strain applied to the cells substrate, not only significantly decreases the dissociation rate of zyxin from the focal adhesion, but also induce focal adhesions stability together with an increase in their size. In both cases, by either growing cells on increasing substrate stiffness or increasing cellular tension by mechanical stretching, zyxin residence time at focal adhesion is found to increase suggesting that higher cellular traction forces correlates with focal adhesion in which zyxin remains bind longer time i.e., is less probable for zyxin to unbind from focal adhesions. In the following section we will explore this hypothesis.

### Cellular traction correlates with the dissociation kinetics of zyxin at cellular and subcellular level

**Fig 3:**
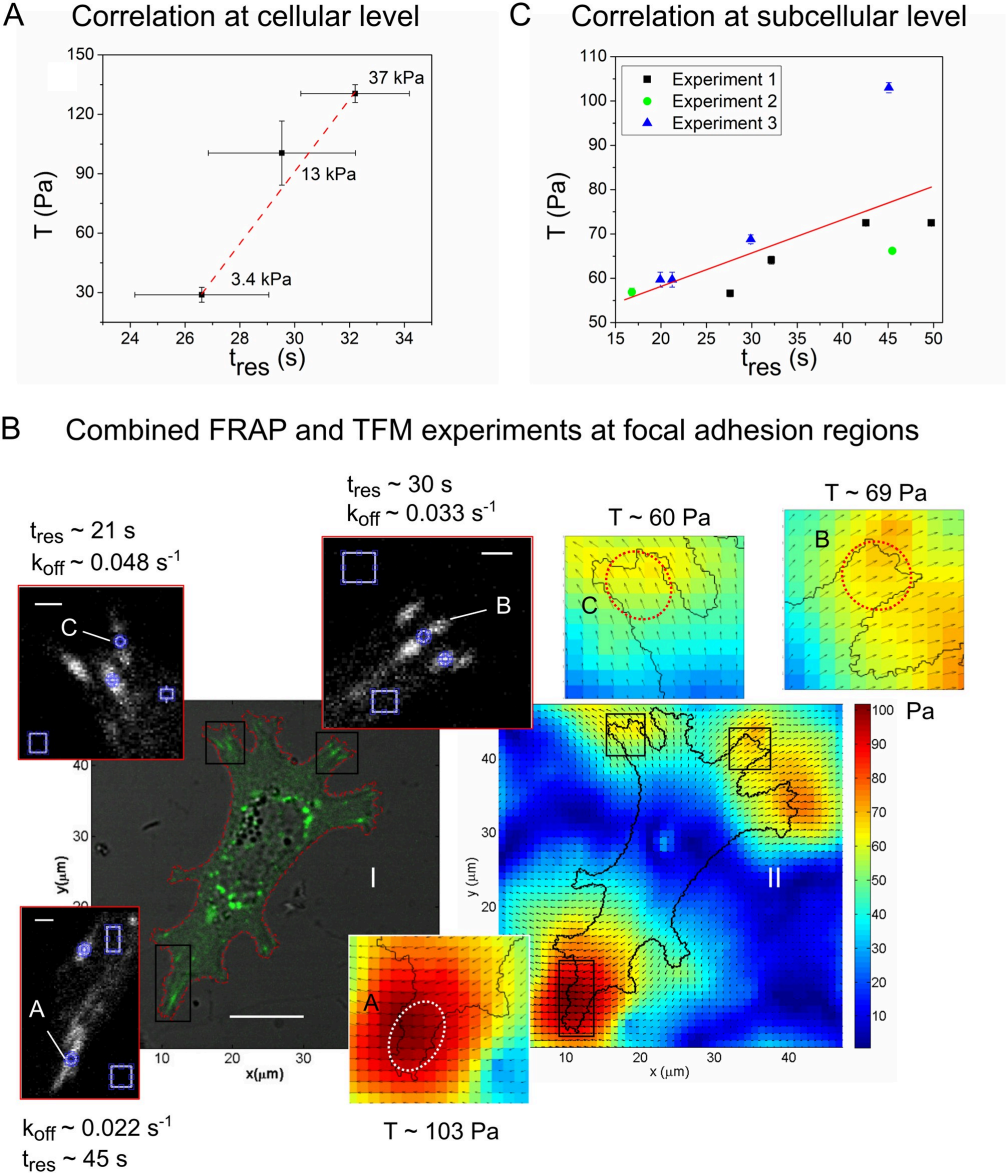
Correlation between cellular traction and residence time of adhesive protein zyxin. **(A) Correlation at cellular level**—different substrate stiffness:correlation between average cellular traction and zyxin average residence time for cells cultivated on different elasticity substrates. Linear fit in dashed line as a guide to the eye. Error bars: SE. **(B) Combined FRAP and TFM experiments at focal adhesions regions: (B.I)** Transmission and fluorescence merged images of a cell expressing zyxin-EGFP cultured on a substrate of 3.4 kPa elasticity (scale bar: 10 μm). The boxes show the zoomed-in fluorescent images of the areas selected for the FRAP analysis (scale bar: 1 μm). The regions considered in the calculation of the recovery curve are marked with circles and rectangles. For each region, the dissociation rate constant, *k*_*off*_, and residence time, *t*_*res*_, of zyxin are presented. **(B.II)** Traction map and zoomed-in images of the selected regions for the calculation of average traction (*T*). **(C) Correlation at subcellular level: focal adhesions regions—**correlation between focal adhesion traction forces, *T*, and zyxin residence time, *t*_*res*_, for three independent combined FRAP and TFM experiments. Average traction was calculated over an area of ~20 μm^2^ in each region where FRAP experiments were performed (note that traction values are higher than those obtained by averaging the whole cell). Linear regression in solid red line.

The results gathered up to now expose a modulation of substrate rigidity on the generation of the traction force and on the dissociation kinetics of zyxin. The estimated dissociation rate constants were then used to calculate the residence time, the inverse of *k*_*off*_. This residence time would represent the average time a zyxin molecule remains at a binding site in the focal adhesion. The results obtained at different substrate mechanical conditions, show an increment of both, the average cell traction and average zyxin residence time as the rigidity of the substrate increases. To further explore this relationship, Fig 3A shows the correlation between the traction forces exerted by cells and the residence time of zyxin at the substrate elasticities studied, suggesting a linear relationship between both magnitudes. Although there is a variation within the residence time for zyxin at a given substrate elasticity (evidenced in a considerable error in the average value), there is a tendency to increase when the substrate elasticity increases. This variability could be due to different magnitude of forces generated at the focal adhesion. Notice that the data presented in Fig 3A is the average residence time for zyxin measured at different focal adhesions, while the average magnitude of traction force is the one produced by the entire cell.

Up to this point the effects of substrate rigidity on the generation of the traction force and on the kinetics of dissociation of the protein zyxin were evaluated independently. These results, would suggest that cells that generate greater traction forces are those that have focal adhesions where is less probable for zyxin to dissociate from. To test this hypothesis, combined TFM and FRAP were performed, addressing the correlation of traction forces exerted by a single cell with the dissociation kinetics of the protein zyxin at a subcellular level of focal adhesions regions. For this purpose, the experimental procedure was slightly modified: after acquiring the marker images for TFM, FRAP experiments were performed in several focal adhesions; then cell-substrate adhesion contacts were removed by trypsinization, and finally another image of the fluorescent markers was acquired.

To estimate traction forces generated by focal adhesion, average cellular traction forces were quantified at different cell regions of the same area with a high presence of focal adhesions. Each of these areas contained a minimum of 3 up to 5 focal adhesions, and the average traction value was determined in an area of ~20 μm^2^. In this way, averaging the traction force of a small region of the cell, dense in focal adhesions, we minimize the error of quantifying the traction force associated to a single focal adhesion (focal adhesion area is ~2.5 μm^2^). We have also seen that neighbour focal adhesions present similar values of zyxin residence time.

Fig 3B shows the results of a representative combined TFM-FRAP experiment performed in a HC11 cell expressing zyxin-EGFP, grown on a substrate of 3.4 kPa. The left panels of Fig 3B show the merged image of the transmission and confocal fluorescent images, where it can be seen that focal adhesions are localized preferably at the periphery of the cell. Three zoomed regions containing focal adhesions were selected for FRAP experiments. Its corresponding traction map is shown on the right in Fig 3B, as well as the enlargements of the regions studied in FRAP. For each selected region, the residence time measured by FRAP and the average focal adhesion traction value over an area of ~20 μm^2^ were estimated, the results of three independent experiments are shown in Fig 3C. For all the experiments, regions that exert greater traction forces correspond to regions with focal adhesions where zyxin has less probability of unbind. To better illustrate this correlation, in Fig 3C is presented the linear regression between traction forces and zyxin characteristic time. Even though there are no variations in the elasticity of the substrate (combined experiments were all performed in 3.4 kPa PAA hydrogels), the analysis of the data indicates a correlation -at focal adhesions regions—between the dissociation kinetics of zyxin and the magnitude of the traction forces exerted by a focal adhesion, suggesting that focal adhesions in which zyxin has less probability of dissociate, would exert greater traction forces.

These results are in agreement with the ones obtained for another mechanosensitive focal adhesion protein, vinculin, in mouse embryonic fibroblasts cultured on 3-, 5- or 14-kPa microfabricated post-array-detectors (mPADs) [29], where vinculin residence time at focal adhesions varied linearly with applied force for stiff substrates, while the residence time at focal adhesions of other adhesive protein, paxillin, was independent of local applied force and substrate stiffness. One disadvantage of mPADS is that cells are presented with topographical cues so their adhesion sites grow on laterally restricted islands, making this system fundamentally different from unconstrained adhesion on flat substrates such as the ones employed here [30]. The direct relationship between zyxin molecular dynamics and traction force generation presented here, not established before, provides further evidence reinforcing the mechanosensitive properties of zyxin, pointing it out as a key protein for cellular traction forces.

## Discussion

In this work, we investigated the effects of substrate stiffness and the correlation between the generation of cellular traction forces and the molecular dynamics of focal adhesion protein zyxin. We explored the relationship between traction forces generated at focal adhesions and the unbinding kinetics of zyxin from focal adhesions. To this end, a combination of quantitative experimental approaches was used. Polyacrylamide hydrogels were fabricated as elastic substrates; by varying the relationship between their components, substrates of different elasticities were obtained ranging from 3.4- to 46- kPa. Through Force Spectroscopy-AFM, the elasticities of the polyacrylamide hydrogels were characterized, and reliable Young’s moduli for the reconstruction of the forces generated by the cells on the hydrogel were obtained. These kinds of unconstrained flat substrates allow a cellular adhesion and spreading closer to physiological in comparison with restricted ones, such as micro post arrays. In the range of elasticity explored, cells were well adhered to the substrate and were able to form focal adhesions.

In order to study the effects of substrate stiffness in the generation of cellular traction forces, we quantified the substrate deformation and traction forces exerted by mouse epithelial mammalian living cells cultivated on polyacrylamide substrates of different elasticity ranging from 3.4- to 37- kPa, by TFM. The analysis of the traction maps indicated that the magnitude of the forces generated by cells increases while cultivating them on more rigid substrates. We found that 46 kPa Young’s modulus substrate was an upper limit for TFM experiments in mouse mammary epithelial HC11 cells, for which substrate deformation due to cellular traction was not detectable.

In particular, we investigated the effects of substrate elasticity on the molecular binding kinetics and diffusion of zyxin, postulated as a mechanosensor protein. To this end, we employed a fluorescent version of zyxin transiently expressed in HC11 cells. A point-FCS analysis allowed us to compare the mobility and possible interactions of zyxin under different mechanical conditions. We did not observe differences in the effective diffusion coefficient, representative of the mobility of the protein *in vivo*, from cells grown on 13 kPa PAA substrates and on coverslips (~70 GPa), suggesting that the mobility of zyxin is not altered by the substrate stiffness. To better assess the molecular binding kinetics of zyxin we characterized the dissociation rate constant of zyxin by means of the FRAP technique, in the same substrate elasticity conditions as the TFM experiments were performed. We found that the dissociation rate constant is modulated by substrate stiffness: the probability of zyxin to unbind from the focal adhesion, decreased as the rigidity of the substrate increases, or in other terms, the residence time for zyxin at the focal adhesion increases. This modulation is in agreement with our previous study in which we observed a modulation in zyxin binding kinetics due to increasing normal strain, employing a mechanical stretching device in living HC11 cells [9]. Thus, zyxin residence time at focal adhesion is increased, either by increasing cellular tension by mechanical stretching or by growing cells on increasing substrate stiffness.

The results obtained from independent TFM and FRAP experiments performed at the same substrate elasticity conditions, revealed a correlation between the average cellular traction and the average residence time for zyxin molecule at the focal adhesion (presented in Fig 3A), indicating that cells exerting greater traction would present longer residence time for zyxin at the focal adhesion. We found however a considerable variation within the residence time for zyxin characterized at a given substrate elasticity in comparison with the variations in the magnitude of traction force produced by the entire cell, probably due to different magnitude of forces generated at single focal adhesions. We further performed combined TFM and FRAP experiments, in which the variation in the traction generated at focal adhesions regions was confirmed. These variations in the traction exerted by individual focal adhesions were concealed by performing the average of the traction exerted throughout the cell.

The ability to measure cellular traction forces together with quantifying focal adhesion molecular dynamics is crucial to better understand the relation between focal adhesion dynamics and cellular traction generation and their relation in substrate rigidity sensing. Therefore, combined single cell experiments of TFM and FRAP allowed us to explore the correlation between the unbinding kinetics of zyxin and the magnitude of the traction forces exerted by a focal adhesion. The analysis of these experiments indicated a linear correlation–at a subcellular level–between the unbinding kinetics of zyxin and the magnitude of the traction forces exerted by focal adhesions: focal adhesions that exerted greater traction forces presented a longer zyxin residence time.

Many of the molecules that constitute focal adhesions are proposed to interact with one another to form an integrated mechanical and biochemical network that regulates the processes involved in cell adhesion and substrate sensing. But the relationship between traction force generation and focal adhesion molecular dynamics is poorly understood, especially regarding the zyxin protein. The relationship between traction force and molecular binding kinetics was previously explored for two other focal adhesion proteins, vinculin and paxillin: while vinculin residence time at focal adhesions was shown to vary linearly with traction force, paxillin residence time was found to be insensitive to the traction [29]. These results are consistent with paxillin not being implicated as a regulator of force transmission, but acting as an important component of the integrin signaling layer [31], and vinculin regulating force transmission between the cell and the extracellular matrix [32]. We have indeed shown in our previous work (Sigaut *et al*., 2018 [9]) that vinculin molecules in living HC11 cells follow the mechanical stretching of the substrate, by increasing their intramolecular tension within focal adhesions. However, the direct relationship between zyxin molecular dynamics and traction force generation was, to our knowledge, not established before.

In conclusion, to reach a better understanding of substrate mechanosensing and the relation between cellular force generation and zyxin molecular dynamics at focal adhesions, we have examined the effect of substrate rigidity at a cellular and subcellular level of focal adhesions regions, by combining live cell imaging, traction force microscopy (TFM), fluorescence recovery after photobleaching (FRAP) and fluorescence correlation spectroscopy (FCS). In addition, we have fabricated adjustable stiffness substrates and characterized their elasticities by Force Spectroscopy-AFM. Using this multidimensional approach, we found a modulation of substrate stiffness on the generation of traction forces and zyxin molecular kinetics, unveiling a positive correlation between the traction forces exerted by cells and the residence time of zyxin at the focal adhesions of HC11 cells. Moreover, combined TFM and FRAP experiments revealed that this correlation endures at the subcellular level, even if there are no variations in substrate stiffness, suggesting that focal adhesions that exert greater traction, present longer residence time for zyxin, i.e., zyxin protein has less probability to dissociate from the focal adhesion. This multidimensional strategy allowed us to address and establish a direct and linear correlation between the generation of traction forces and the molecular dynamics of protein zyxin at focal adhesions regions. These results provide further evidence reinforcing the mechanosensitive properties of zyxin and its key role in cellular traction forces.

## Materials and methods

### Activation of the coverslips surface

To ensure tight attachment of the PAA hydrogel to the glass surface, circular glass coverslips of 25 mm or 12 mm diameter were chemically modified to allow covalent attachment of polyacrylamide substrates using previously developed protocol [10]. Briefly, coverslips previously cleaned with 0.1% HCl and ethanol were first submersed in a solution of 0.5% (v/v) 3-aminopropyltrimethoxysilane (Sigma-Aldrich, St. Louis, MO) in ethanol for 5 minutes and washed with ethanol. Then, were treated with a solution of 0.5% glutaraldehyde (Fluka^®^ Analytical) in deionized water for 30 minutes, washed with deionized water and left to dry.

### Fabrication of polyacrylamide substrates

To produce PAA hydrogels, stock solutions of 40% acrylamide (Sigma-Aldrich, St. Louis, MO) and 2% bis-acrylamide (Sigma-Aldrich) were combined, following the previously developed protocol [6]. Depending on the mole fraction of acrylamide and bis-acrylamide, substrates with elastic moduli between 3.4 kPa and 46 kPa were obtained (see Table 1). Crimson fluorescent nanospheres of 40 nm diameter (carboxylate-modified, Ex/Em 625/645, Thermo Fisher Scientific, Waltham, MA), were thoroughly mixed with the polyacrylamide solution (0.004% v/v). The polymerization was initiated by incorporating N,N,N,N-tetramethylethylenediamine (TEMED, Sigma-Aldrich) and ammonium persulfate 10% solution (APS, Bio-Rad Laboratories, Inc., Hercules, CA). Droplets of 10 μl of the final solution were placed on top of activated circular coverslips (25 mm diameter) and then carefully covered with untreated coverslips (12 mm diameter) to produce flatten PAA hydrogels of approximately 50 μm thickness. The thickness of the substrate was measured by confocal microscopy. Following polymerization, the untreated coverslips were mechanically detached and the PAA substrates were always maintained hydrated.

### Characterization of nanomechanical properties of PAA hydrogels

To determine nanomechanical properties of PAA hydrogels, Force Spectroscopy-AFM was performed by an atomic force microscope. Hydrogels of 50 μm thickness were prepared on activated circular coverslips of 12 mm diameter and were always kept in optimal conditions of humidity. Force Spectroscopy-AFM experiments were performed with a Bruker Multimode 8 AFM with a Nanoscope V controller (Bruker, Santa Barbara, CA, USA) employing a sharp nitride lever AFM probe (SNL, Bruker), with nominal values of 2 nm tip radius, 0.06 N/m spring constant and 18 kHz resonant frequency. Before each experiment, the spring constant was calibrated using the thermal tune method [33]. The AFM tip radius was measured using the standard polycrystalline titanium roughness sample provided by Bruker and the Tip Qualification function in NanoScope Analysis software. The deflection sensitivity was determined using a hard sample (sapphire sample provided by Bruker).

Force curves were acquired in solution (PBS, Phosphate Buffered Saline) distributed in different regions of PAA hydrogels. The contact point of each individual curve was determined according to a published algorithm [34]. Young’s modulus (*E*) was estimated from the approach force-distance curves by fitting using the Sneddon model [35, 36]:

$$F=\frac{2}{\pi }\cdot \frac{E}{1-\nu^{2}}\cdot tan\left(\alpha \right)\cdot \delta^{2}$$ (1)

where *F* is the force, δ is the indentation, α the tip half open angle and ν the Poisson’s ratio (usually assumed to be 0.5 for such hydrogels).

Hundreds of force curves were analyzed using a custom routine written in Matlab (MathWorks Inc., Natick, USA) platform to select the elastic regime of the approach force distance curves and then fitted by the Sneddon model. S1 Fig shows representative curves for 4 different elasticity substrates and histograms showing the distribution of Young’s modulus. The values obtained are shown in Table 1. In the case of the softest PAA hydrogel (hydrogel A), the difficulty to determine the point of contact in the force-distance curves lead to a great variability in Young’s modulus, so the measured values were not reliable. In such a case, Young’s modulus was estimated by proportion of its constituents taking the values from Aratyn-Schaus *et al*., 2010 [10] and, using as a reference the closest Young’s modulus value obtained for the measured hydrogel, see Table 1.

### Functionalization of polyacrylamide substrates

To compatibilize PAA substrates with cell culture and promote cell attachment, PAA hydrogels were sterilized with UV irradiation and coated with the extracellular matrix protein fibronectin (50 μg/ml, Sigma-Aldrich, St. Louis, MO) using the bifunctional crosslinker sulfo-SANPAH (Pierce Biotechnology, Rockford, IL).

### Cell culture and transfection

The HC11 cell line, derived from pregnant BALB/c mouse mammary glands [37], were grown under controlled CO_2_ atmosphere at 37 °C in RPMI 1640 medium (Invitrogen–Gibco, Thermo Fisher Scientific Inc, Waltham, MA) supplemented with antibiotic-antimycotic (Invitrogen—Gibco), 10% fetal calf serum and 5 μg per ml of insulin from bovine pancreas (Sigma Aldrich, St. Louis, MO). In order to visualize focal adhesions via the molecular scaffold protein zyxin, HC11 cells were transiently transfected using Lipofectamine^®^ 2000 Reagent (Invitrogen) employing the zyxin-EGFP construction (courtesy of Dr. D. E. Ingber). Transfected HC11 cells were allowed to grow for 24 h and then were seeded on top of the fibronectin-coated PAA hydrogels, and cultured for 12–24 hours to let them completely adhere.

### Microscopy—Image acquisition

Live-cell microscopy was performed in a spectral confocal scanning microscope FluoView 1000 (Olympus Co., Japan), employing a 60X UPLSAPO oil immersion objective lens NA 1.35. A microscope incubator chamber was used to maintain 37°C temperature during experiments. Images were obtained using a pixel size between 33 nm and 69 nm, and the time per pixel between 10 μs and 20 μs. For TFM, Crimson nanospheres were monitored with a 635 nm diode laser and the emitted fluorescence was detected in the [655–755] nm range. FRAP and FCS experiments were performed between 24 and 48 hours after transfection. For monitoring EGFP the 488 nm line of the Argon laser was used, and the emitted fluorescence was detected at [500–550] nm. In combined TFM-FRAP experiments, images were acquired in a sequential mode, to avoid crosstalk between channels.

### TFM experiment acquisition and image selection

TFM experiments were performed in HC11 cells cultivated on fibronectin coated PAA substrates of 3.4 -, 13 -, 37—and 46—kPa, maintained at 37 °C. Isolated cells -separated at least 15 μm from other cells- were selected, so that the deformations generated by neighboring cells do not interfere with cellular traction quantification. Confocal sequence images of Crimson nanospheres at different depths (PRE *z*-stack) were acquired, between 3 to 5 planes from the substrate surface to the interior, in 0.5 μm steps. Images were obtained using Kalman filter, employing image sizes 1024x1024 or 1600x1600 pixels depending on cell size and maintaining pixel size smaller than 69 nm. Cells were detached from the substrate by trypsinization. The time needed to separate the cells from the substrate, and allow the substrate to relax was between 15–20 min. After cell detachment, another *z*-stack image was acquired (POST). For possible stage drift correction, PRE and POST *z*-stack images were aligned employing Template Matching plugin (ImageJ, NIH) [38], using as a reference a region not affected by cellular traction (typically on the edge of the image). The optimal planes for TFM analysis were selected, choosing the pair of PRE and POST planes that presented maximum nanospheres displacement.

### Deformation maps

Extraction of a discrete displacement field describing the deformation of PAA substrates was done by analyzing Crimson nanospheres images before (PRE) and after detachment (POST) of the adhering cell with trypsin. The positions of the markers were tracked using the particle image velocimetry (PIV) algorithm employing the open-source Matlab code MatPIV V1.6.1 of Sveen, 2004 [18]. This algorithm is based on cross-correlating image sub-regions between sequential pairs of images. By processing the images over a regular grid of small sub-regions, a displacement vector map is generated. The algorithm was configured to start with large interrogation windows 256x256 or 128x128 pixels (around 10 μm^2^) whose size was reduced, in powers of 2, to obtain windows of approximately 32x32 or 16x16 pixels (1 μm^2^). In order to reduce the distance between the interrogation windows, an overlap of 50 percent was used. To remove spurious vectors (in general due to image sub-regions with not enough or too many particles to create a good pattern for matching), a series of filters are applied: a signal-to-noise ratio filter, a global histogram filter, a local filter and a masking mode for neglecting regions without out fluorescent markers. Finally, all the identified outliers are interpolated using a nearest neighbor interpolation. The deformation map shows in each window the vector of the deformation obtained by locating the peaks of the cross correlation between the PRE and POST images of each interrogation window. The color code represents the module of the intensity of the deformation.

### Traction force reconstruction and quantification

Traction force fields were reconstructed from the deformation fields considering PAA substrates as an elastic, isotropic and semi-infinite material, and substrate deformations mainly contained in the plane. For traction estimation, the Young’s modulus and Poisson’s ratio are required. Young’s modulus of the substrate was determined experimentally by Force Spectroscopy-AFM and its Poisson’s ratio was considered equal to 0.5. Traction force reconstruction was accomplished by applying Fourier Transform Traction Cytometry, employing an adapted routine in Matlab platform based on FTTC implementation of Sabass *et al*., 2008 [19]. As this is an ill-posed inverse problem, the Tikhonov regularization method [20] was used incorporating a parameter of regularization, this method efficiently produces a continuous and smooth reconstructed traction field. To select the optimal value of the regularization parameter in an objective manner, the L-Curve criterion was used [21], considering the point of maximum curvature as the *optimal regularization value*.

In order to extract information concerning to the cell, a mask was manually defined by selecting the cell area from the transmission image, or automatically detected in the zyxin-EGFP fluorescent image in combined TFM-FRAP experiments. The average magnitude values of deformation (*D*) and cellular traction (*T*) were estimated by applying the cell mask on the deformation and traction map, respectively, and then the average cell force magnitude (*F*) was calculated by multiplying the average traction by the cell area. Also, the histograms of the module of each component of the measured magnitudes can be made. S2C Fig shows the cell mask applied to the traction field, which was used to calculate the average value of the traction magnitude exerted by the cell. The histograms of the magnitude and components of cellular traction and deformation field are presented in S2D Fig.

### FCS experiments and analysis

FCS experiments were performed in HC11 cells expressing zyxin-EGFP cultivated on fibronectin coated PAA substrates of 13 kPa and on fibronectin coated coverslips. For single-point FCS, fluorescence intensity over a single point within a focal adhesion, *I(t)*, was recorded as a function of time for 1.5 minutes, at a 100 kHz sampling rate. The acquisition was performed in the pseudo-photon counting mode, and the fluorescence was detected over [500–600] nm range. The normalized autocorrelation function of the fluorescence intensity fluctuation, δ*I(t)*, around its mean value 〈*I(t)*〉 is defined as

$$G\left(\tau \right)=\frac{\left\langle \delta I\left(t\right)\cdot \delta I\left(t+\tau \right)\right\rangle }{{\left\langle I\left(t\right)\right\rangle }^{2}}.$$ (2)

Autocorrelation curves were calculated with a custom-made routine written on the Matlab platform. Every record was divided into 1021 sub-records of 213 data-points that were subsequently doubled with zeros to avoid aliasing effects. The autocorrelation curve of each of these sub-records were computed and then averaged. The averaged auto-correlation curves were fitted using a 1- or 2- component model [22], with i = 1 and i = 1, 2, respectively:

$$G(\tau )=\sum_{i=1,2}G_{oi}\cdot {\left(1+\frac{\tau }{\tau_{Di}}\right)}^{-1}\cdot {\left(1+\frac{\tau }{\omega^{2}\tau_{Di}}\right)}^{-1/2}$$ (3)

where ω = ω_z /_ ω_r_ is the ratio of axial (ω_z_) to radial (ω_r_) dimensions of the detection volume (assuming a Gaussian volume), *G*_*oi*_, is the autocorrelation amplitude and the characteristic correlation time, τ_*Di*_. This time depends on the diffusion coefficient of the molecule, *D*_*i*_, and on the size of the detection volume, and is defined as

$$\tau_{Di}=\frac{w_{r}^{2}}{4D_{i}}.$$ (4)

Prior to estimating a diffusion coefficient from the characteristic time, calibration experiments were performed to determine the geometric parameters of the detection volume (ω_z_ and ω_r_), employing an aqueous solution of a known diffusion coefficient (in this case, fluorescein, with a diffusion coefficient of 425 μm^2^/s [39]).

### FRAP experiments and analysis

FRAP experiments were performed in HC11 cells expressing zyxin-EGFP cultivated on fibronectin coated PAA hydrogels of 3.4-, 13- and 37- kPa. A selected small region (0.2–0.5) μm^2^ within a focal adhesion is photobleached employing nominal 100% transmission of the 488 nm line from an Argon laser (approximately 0.5 mW) during 40–200 ms. Before bleaching, between 5 to 10 control images are taken and immediately after bleaching a sequence of images is captured every 1 second during the first 40 s, and every 4 s during the last 40 s. Employing a custom-made Matlab routine, the mean fluorescence intensity within the selected bleached region is calculated as a function of time, *I*_*PH-FA*_. To minimize the effect of photobleaching during acquisition, *I*_*PH-FA*_, was divided by the mean fluorescence intensity of a control region of the same area (in a focal adhesion not photobleached), *I*_*C-FA*_, obtaining

$$I_{FRAP}(t)=\frac{I_{PH-FA}(t)-I_{Cyt}}{I_{C-FA}(t)-I_{Cyt}}$$ (5)

where *I*_*Cyt*_ is the background intensity calculated as the mean fluorescence intensity of a cytoplasmic region. Then the recovery curve is calculated as:

$$F(t)=\frac{I_{FRAP}(t)-I_{o}}{I_{PRE}-I_{o}}$$ (6)

where *I*_*PRE*_ is the intensity in the bleached region before photobleaching, *I*_*o*_ is the intensity in the bleached region immediately after photobleaching. An example of the selected regions and the fluorescence intensity on background (*I*_*Bkg*_), cytoplasm (*I*_*Cyt*_), control adhesion (*I*_*C-FA*_) and photophotobleached adhesion (*I*_*PH-FA*_), together with recovery curve, are shown in S3 Fig.

Assuming that diffusion of unbound zyxin in the cytoplasm is much faster than its binding/unbinding kinetics to the focal adhesion, and photobleaching of cytoplasmic protein is negligible, the recovery during FRAP is purely due to recovery in the bound concentration of protein to the focal adhesion [27, 28, 40]. When the dissociation kinetics from the focal adhesion is rate limiting, the unbinding rate constant of zyxin, *k*_*off*_, is determined by fitting (least-squares best fit) FRAP recovery data to the expression [28]:

$$F(t)=m\cdot \left(1-e^{-k_{off}\cdot t}\right)$$ (7)

where *m* is the mobile fraction. From the dissociation rate constant, the average time a molecule remains at a binding site, *t*_*res*_, namely the residence time, can be estimated as:

$$t_{res}=\frac{1}{k_{off}}.$$ (8)

## Supporting information

S1 Fig Characterization of nanomechanical properties of PAA hydrogels by Force Spectroscopy-AFM. (A) Representative approach force-distance curves for different elasticity substrates, 6 curves for each PAA hydrogel are shown. (B) Histograms showing the distribution of Young’s modulus of PAA hydrogel estimated from the approach force-distance curves by using the Sneddon model. The composition of Acrylamide/Bis-acrylamide of fabricated Polyacrylamide substrates is presented in Table 1.
(TIF)

S2 Fig TFM experiments to quantify cellular traction of cells cultivated on PAA substrates containing fluorescent nanospheres. **(A)** Representative transmission image (center column) and deformation (left column) and traction (right column) maps for a HC11 cell grown on a 13 kPa PAA substrate. The border of the cell is delimited by red dashed lines in each image, scale bar 10 μm. **(B)** Merge confocal image of fluorescent nanospheres distribution before (red) and after (green) detaching the cell (center column) of the selected region in (A), scale bar 10 μm. Zoomed-in image of the deformation (left column) and traction (right column) maps, where vectors indicate the direction, and their magnitudes are given by a color-coded scale. **(C)** Cell mask applied to the traction map to calculate the average value of the traction magnitude exerted by the cell. **(D)** Histograms of the module of each direction (Ux and Uy) and magnitude (|U|) of the substrate deformation and histograms of the magnitude (|T|) and components (Tx and Ty) of cellular traction.
(TIF)

S3 Fig FRAP experiment in HC11 cell cultivated on PAA substrate to evaluate zyxin unbinding kinetics. **(A)** Representative region of a confocal image of a cell expressing zyxin-EGFP, scale bar 1 μm. Selected regions to quantify fluorescence intensity on background (Bkg), cytoplasm (Cyt), control focal adhesion (C-FA) and photobleached focal adhesion (Ph-FA). **(B)** Fluorescence intensity at regions selected in (A). Photobleaching is indicated with the vertical dotted line at t = 0. **(C)** Fluorescence recovery curve for zyxin (black line) calculated according to Eqs 5 and 6, and fitting by Eq 7 (red line).
(TIF)

S1 Table Zyxin dynamics evaluated by pointFCS in HC11 cells cultivated on 13 kPa PAA substrates and on coverslips. Fitting parameters for FCS experiments, by 1- or 2- component model, Eq 3. Average autocorrelation amplitude, *G*_*oi*_, and diffusion coefficient, *D*_*i*_, obtained from data fitting of N experiments. * Two component model fit gives the same diffusive component (*D*_*1*_ = *D*_*2*_).
(PDF)

## References

[1] Geiger B, Spatz JP, Bershadsky AD. Environmental sensing through focal adhesions. Nat Rev Mol Cell Bio. 2009;10(1):21–33. 10.1038/nrm2593 19197329

[2] Sunyer R, Jin AJ, Nossal R, Sackett DL. Fabrication of hydrogels with steep stiffness gradients for studying cell mechanical response. PLoS ONE. 2012;7(10):e46107–e. 10.1371/journal.pone.0046107 . 23056241 PMC3464269

[3] Fu J, Wang Y-K, Yang MT, Desai RA, Yu X, Liu Z, et al. Mechanical regulation of cell function with geometrically modulated elastomeric substrates. Nat Methods. 2010;7(9):733–6. http://www.nature.com/nmeth/journal/v7/n9/abs/nmeth.1487.html#supplementary-information 20676108 10.1038/nmeth.1487 PMC3069358

[4] Plodinec M, Loparic M, Monnier CA, Obermann EC, Zanetti-Dallenbach R, Oertle P, et al. The nanomechanical signature of breast cancer. Nat Nanotech. 2012;7:757. https://www.nature.com/articles/nnano.2012.167#supplementary-information. 23085644 10.1038/nnano.2012.167

[5] Vogel V, Sheetz M. Local force and geometry sensing regulate cell functions. Nat Rev Mol Cell Bio. 2006;7(4):265–75. 10.1038/nrm1890 16607289

[6] Zaidel-Bar R, Ballestrem C, Kam Z, Geiger B. Early molecular events in the assembly of matrix adhesions at the leading edge of migrating cells. J Cell Sci. 2003;116(22):4605. 14576354 10.1242/jcs.00792

[7] Lele TP, Pendse J, Kumar S, Salanga M, Karavitis J, Ingber DE. Mechanical forces alter zyxin unbinding kinetics within focal adhesions of living cells. J Cell Physiol. 2006;207(1):187–94. 10.1002/jcp.20550 16288479

[8] Colombelli J, Besser A, Kress H, Reynaud EG, Girard P, Caussinus E, et al. Mechanosensing in actin stress fibers revealed by a close correlation between force and protein localization. J Cell Sci. 2009;122(Pt 10):1665–79. 10.1242/jcs.042986 19401336

[9] Sigaut L, von Bilderling C, Bianchi M, Burdisso JE, Gastaldi L, Pietrasanta LI. Live cell imaging reveals focal adhesions mechanoresponses in mammary epithelial cells under sustained equibiaxial stress. Sci Rep. 2018;8(1):9788. 10.1038/s41598-018-27948-3 29955093 PMC6023913

[10] Aratyn-Schaus Y, Oakes PW, Stricker J, Winter SP, Gardel ML. Preparation of complaint matrices for quantifying cellular contraction. JoVE. 2010;(46):2173. 10.3791/2173 . 21178972 PMC3159639

[11] Beningo KA, Wang Y-L. Flexible substrata for the detection of cellular traction forces. Trends Cell Biol. 2002;12(2):79–84. 10.1016/s0962-8924(01)02205-x 11849971

[12] Califano JP, Reinhart-King CA. Substrate Stiffness and Cell Area Predict Cellular Traction Stresses in Single Cells and Cells in Contact. Cell Mol Bioeng. 2010;3(1):68–75. 10.1007/s12195-010-0102-6 . 21116436 PMC2992361

[13] Han SJ, Bielawski KS, Ting LH, Rodriguez ML, Sniadecki NJ. Decoupling substrate stiffness, spread area, and micropost density: a close spatial relationship between traction forces and focal adhesions. Biophys J. 2012;103(4):640–8. 10.1016/j.bpj.2012.07.023 . 22947925 PMC3443781

[14] Scott LE, Mair DB, Narang JD, Feleke K, Lemmon CA. Fibronectin fibrillogenesis facilitates mechano-dependent cell spreading, force generation, and nuclear size in human embryonic fibroblasts. Integr Biol (Camb). 2015;7(11):1454–65. 10.1039/c5ib00217f . 26412391 PMC4630078

[15] Kraning-Rush CM, Califano JP, Reinhart-King CA. Cellular traction stresses increase with increasing metastatic potential. PLoS ONE. 2012;7(2):e32572–e. 10.1371/journal.pone.0032572 . 22389710 PMC3289668

[16] McKenzie AJ, Hicks SR, Svec KV, Naughton H, Edmunds ZL, Howe AK. The mechanical microenvironment regulates ovarian cancer cell morphology, migration, and spheroid disaggregation. Sci Rep. 2018;8(1):7228. 10.1038/s41598-018-25589-0 29740072 PMC5940803

[17] Engler AJ, Rehfeldt F, Sen S, Discher DE. Microtissue Elasticity: Measurements by Atomic Force Microscopy and Its Influence on Cell Differentiation. Methods Cell Biol. 83: Academic Press; 2007. p. 521–45. 10.1016/S0091-679X(07)83022-6 17613323

[18] Sveen JK. An introduction to MatPIV v. 1.6.1., Department of Mathematics, University of Oslo 2004. 1–27 p.

[19] Sabass B, Gardel ML, Waterman CM, Schwarz US. High resolution traction force microscopy based on experimental and computational advances. Biophys J. 2008;94(1):207–20. Epub 2007/09/07. 10.1529/biophysj.107.113670 . 17827246 PMC2134850

[20] Tikhonov A. Solution of Incorrectly Formulated Problems and the Regularization Method. Soviet Math Dokl. 1963;5:1035/8.

[21] Hansen P. The L-curve and its use in the numerical treatment of inverse problems. Computational Inverse Problems in Electrocardiology. Adv Comp Bioeng. 2001;5:119.

[22] Digman MA, Brown CM, Horwitz AR, Mantulin WW, Gratton E. Paxillin dynamics measured during adhesion assembly and disassembly by correlation spectroscopy. Biophys J. 2008;94(7):2819–31. Epub 2007/11/09. 10.1529/biophysj.107.104984 . 17993500 PMC2267137

[23] Digman MA, Wiseman PW, Horwitz AR, Gratton E. Detecting protein complexes in living cells from laser scanning confocal image sequences by the cross correlation raster image spectroscopy method. Biophys J. 2009;96(2):707–16. 10.1016/j.bpj.2008.09.051 . 19167315 PMC2716688

[24] Hoffmann J-E, Fermin Y, Stricker RL, Ickstadt K, Zamir E. Symmetric exchange of multi-protein building blocks between stationary focal adhesions and the cytosol. eLife. 2014;3:e02257–e. 10.7554/eLife.02257 . 24894463 PMC4040925

[25] Wolfenson H, Bershadsky A, Henis YI, Geiger B. Actomyosin-generated tension controls the molecular kinetics of focal adhesions. J Cell Sci. 2011;124(9):1425. 10.1242/jcs.077388 21486952 PMC3078811

[26] Lele TP, Thodeti CK, Ingber DE. Force meets chemistry: Analysis of mechanochemical conversion in focal adhesions using fluorescence recovery after photobleaching. J Cell Biochem. 2006;97(6):1175–83. 10.1002/jcb.20761 16408278

[27] Sprague BL, Pego RL, Stavreva DA, McNally JG. Analysis of Binding Reactions by Fluorescence Recovery after Photobleaching. Biophys J. 2004;86(6):3473–95. 10.1529/biophysj.103.026765 15189848 PMC1304253

[28] Lele T, Oh P, Nickerson J, Ingber DE. An improved mathematical approach for determination of molecular kinetics in living cells with FRAP. Mech Chem Biosyst. 2004;1(3):181–90. 16783931

[29] Zhou DW, Lee TT, Weng S, Fu J, García AJ. Effects of substrate stiffness and actomyosin contractility on coupling between force transmission and vinculin-paxillin recruitment at single focal adhesions. Mol Biol Cell. 2017;28(14):1901–11. 10.1091/mbc.E17-02-0116 . 28468976 PMC5541841

[30] Schwarz US, Soiné JRD. Traction force microscopy on soft elastic substrates: A guide to recent computational advances. BBA-Mol Cell Res. 2015;1853(11, Part B):3095–104. 10.1016/j.bbamcr.2015.05.028 26026889

[31] Kanchanawong P, Shtengel G, Pasapera AM, Ramko EB, Davidson MW, Hess HF, et al. Nanoscale architecture of integrin-based cell adhesions. Nature. 2010;468(7323):580–4. 10.1038/nature09621 . 21107430 PMC3046339

[32] Dumbauld DW, Lee TT, Singh A, Scrimgeour J, Gersbach CA, Zamir EA, et al. How vinculin regulates force transmission. Proc Natl Acad Sci U S A. 2013;110(24):9788–93. Epub 2013/05/28. 10.1073/pnas.1216209110 . 23716647 PMC3683711

[33] Hutter JL, Bechhoefer J. Calibration of atomic‐force microscope tips. Rev Sci Instrum. 1993;64(7):1868–73. 10.1063/1.1143970

[34] Lin DC, Dimitriadis EK, Horkay F. Robust Strategies for Automated AFM Force Curve Analysis—I. Non-adhesive Indentation of Soft, Inhomogeneous Materials. J Biomech Eng. 2006;129(3):430–40. 10.1115/1.2720924 17536911

[35] Sneddon IN. The relation between load and penetration in the axisymmetric boussinesq problem for a punch of arbitrary profile. J Biomed Eng. 1965;3(1):47–57. 10.1016/0020-7225(65)90019-4.

[36] McConney ME, Singamaneni S, Tsukruk VV. Probing Soft Matter with the Atomic Force Microscopies: Imaging and Force Spectroscopy. Polym Rev. 2010;50(3):235–86. 10.1080/15583724.2010.493255

[37] Hynes NE, Taverna D, Harwerth IM, Ciardiello F, Salomon DS, Yamamoto T, et al. Epidermal growth factor receptor, but not c-erbB-2, activation prevents lactogenic hormone induction of the beta-casein gene in mouse mammary epithelial cells. Mol Cell Biol. 1990;10(8):4027–34. 10.1128/mcb.10.8.4027 2196443 PMC360913

[38] Schindelin J, Arganda-Carreras I, Frise E, Kaynig V, Longair M, Pietzsch T, et al. Fiji: an open-source platform for biological-image analysis. Nat Methods. 2012;9:676. https://www.nature.com/articles/nmeth.2019#supplementary-information. 22743772 10.1038/nmeth.2019 PMC3855844

[39] Culbertson CT, Jacobson SC, Michael Ramsey J. Diffusion coefficient measurements in microfluidic devices. Talanta. 2002;56(2):365–73. 10.1016/S0039-9140(01)00602-6 18968508

[40] Kaufman EN, Jain RK. Quantification of transport and binding parameters using fluorescence recovery after photobleaching. Potential for in vivo applications. Biophys J. 1990;58(4):873–85. 10.1016/S0006-3495(90)82432-2 2248992 PMC1281033

