# Supplementary figures and images for: Correlation of cellular traction forces and dissociation kinetics of adhesive protein zyxin revealed by multi-parametric live cell microscopy
Source: PLoS One. 2021 May 11;16(5):e0251411. doi: 10.1371/journal.pone.0251411 (PMC8112686; doi:10.1371/journal.pone.0251411)

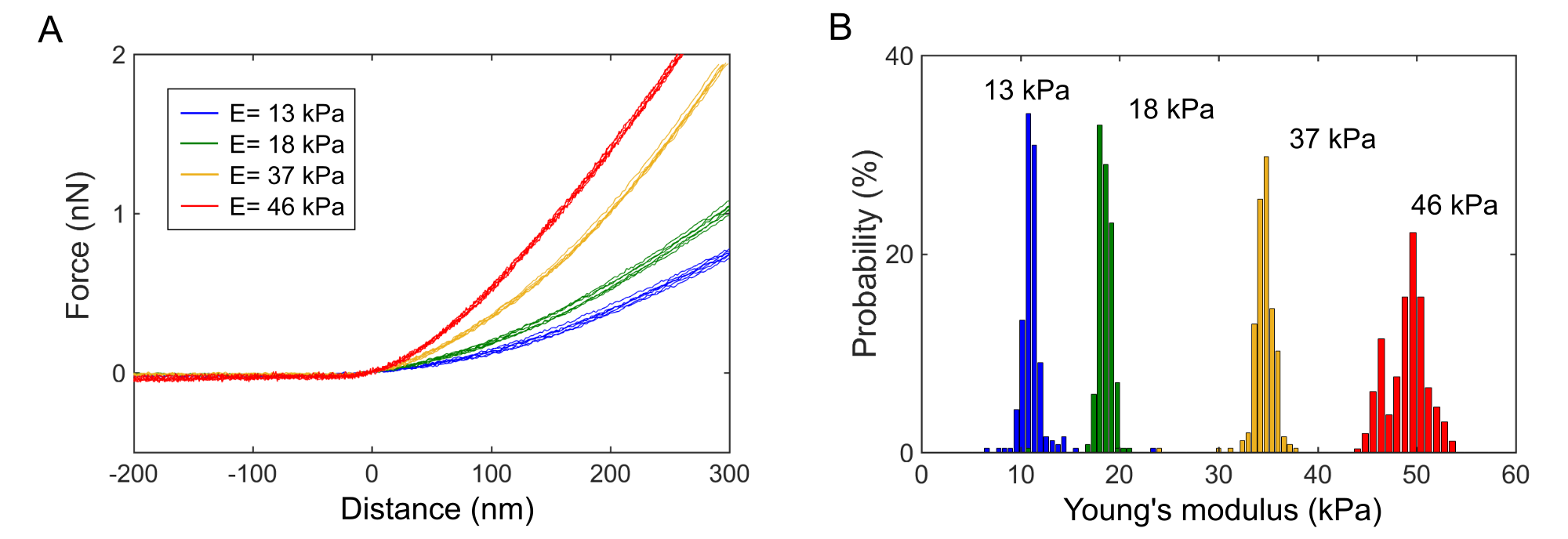

Supplement: S1 Fig — (A) Representative approach force-distance curves for different elasticity substrates, 6 curves for each PAA hydrogel are shown. (B) Histograms showing the distribution of Young’s modulus of PAA hydrogel estimated from the approach force-distance curves by using the Sneddon model. The composition of Acrylamide/Bis-acrylamide of fabricated Polyacrylamide substrates is presented in Table 1. (TIF) [file pone.0251411.s001.tif]

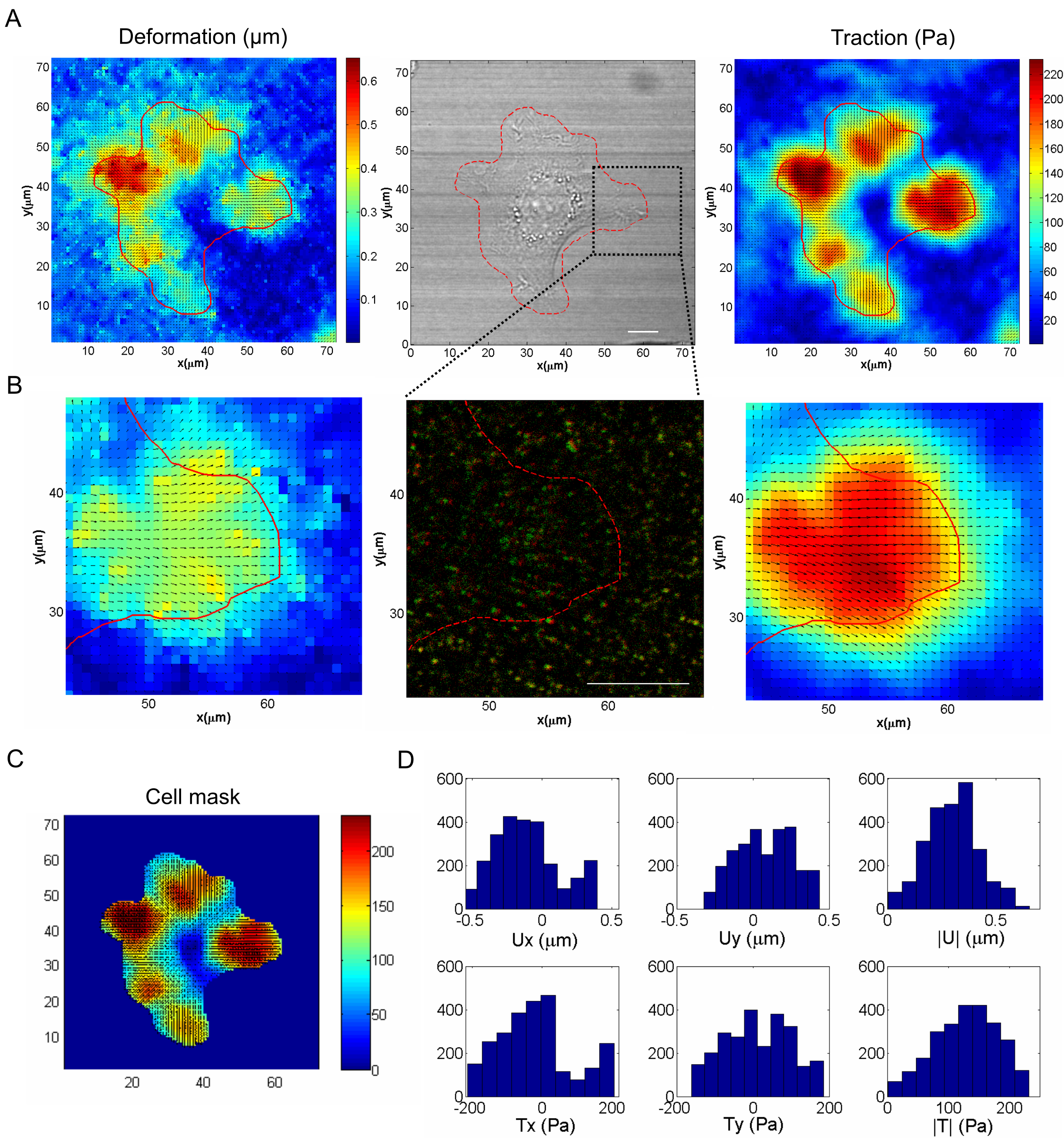

Supplement: S2 Fig — (A) Representative transmission image (center column) and deformation (left column) and traction (right column) maps for a HC11 cell grown on a 13 kPa PAA substrate. The border of the cell is delimited by red dashed lines in each image, scale bar 10 μm. (B) Merge confocal image of fluorescent nanospheres distribution before (red) and after (green) detaching the cell (center column) of the selected region in (A), scale bar 10 μm. Zoomed-in image of the deformation (left column) and traction (right column) maps, where vectors indicate the direction, and their magnitudes are given by a color-coded scale. (C) Cell mask applied to the traction map to calculate the average value of the traction magnitude exerted by the cell. (D) Histograms of the module of each direction (Ux and Uy) and magnitude (|U|) of the substrate deformation and histograms of the magnitude (|T|) and components (Tx and Ty) of cellular traction. (TIF) [file pone.0251411.s002.tif]

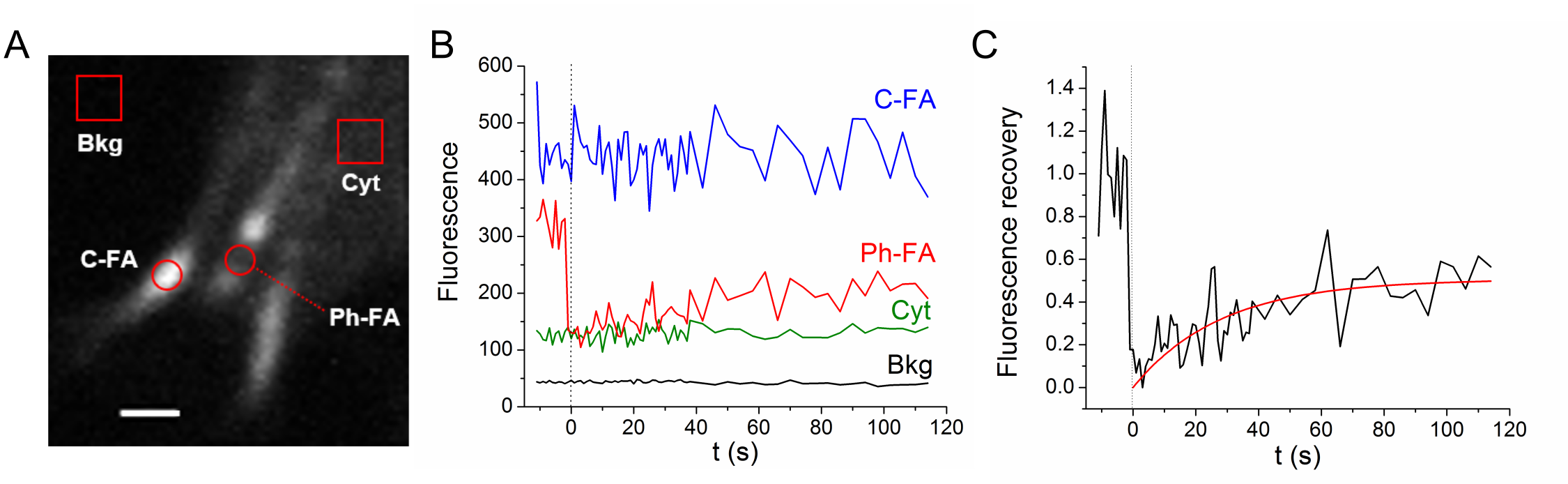

Supplement: S3 Fig — (A) Representative region of a confocal image of a cell expressing zyxin-EGFP, scale bar 1 μm. Selected regions to quantify fluorescence intensity on background (Bkg), cytoplasm (Cyt), control focal adhesion (C-FA) and photobleached focal adhesion (Ph-FA). (B) Fluorescence intensity at regions selected in (A). Photobleaching is indicated with the vertical dotted line at t = 0. (C) Fluorescence recovery curve for zyxin (black line) calculated according to Eqs 5 and 6, and fitting by Eq 7 (red line). (TIF) [file pone.0251411.s003.tif]
