# Supplementary material for: Correlation of cellular traction forces and dissociation kinetics of adhesive protein zyxin revealed by multi-parametric live cell microscopy
Source: PLoS One. 2021 May 11;16(5):e0251411. doi: 10.1371/journal.pone.0251411 (PMC8112686; doi:10.1371/journal.pone.0251411)
Supplement: S1 Table — Fitting parameters for FCS experiments, by 1- or 2- component model, Eq 3. Average autocorrelation amplitude, Goi, and diffusion coefficient, Di, obtained from data fitting of N experiments. * Two component model fit gives the same diffusive component (D1 = D2). (PDF) [file pone.0251411.s004.pdf]

S1 Table. Zyxin dynamics evaluated by pointFCS in HC11 cells cultivated on 13 kPa PAA substrates and on coverslips.

	N	D_1 (µm²/s)	G_{o1}	D_2 (µm²/s)	G_{o2}
**13 kPa PAA**	11				
1-component		11.5 ± 3.3	0.00046 ± 0.00003		
2-components		11.5 ± 3.3	0.00046 ± 0.00003	*	-
**Coverslips**	8				
1-component		11.7 ± 1.1	0.00195 ± 0.00005		
2-components		15.8 ± 3.3	0.0018 ± 0.0001	0.34 ± 0.90	0.0004 ± 0.0025

Fitting parameters for FCS experiments, by 1- or 2- component model, Eq. 3. Average autocorrelation amplitude, G_{oi}, and diffusion coefficient, D_i, obtained from data fitting of N experiments. * Two component model fit gives the same diffusive component ($D_1 = D_2$).
